# Accounting for complex intracluster correlations in longitudinal cluster randomized trials: a case study in malaria vector control

**DOI:** 10.1186/s12874-023-01871-2

**Published:** 2023-03-17

**Authors:** Yongdong Ouyang, Manisha A. Kulkarni, Natacha Protopopoff, Fan Li, Monica Taljaard

**Affiliations:** 1 Clinical Epidemiology Program, Ottawa Hospital Research Institute, 1053 Carling Ave, Ottawa, ON K1Y 4E9 Canada; 2 School of Epidemiology and Public Health, University of Ottawa, 600 Peter Morand Crescent, Ottawa, ON Canada; 3 Department of Disease Control, London School of Hygiene & Tropical Medicine, London, UK; 4 Department of Biostatistics, Yale School of Public Health, New Haven, CT USA; 5 Center for Methods in Implementation and Prevention Science, Yale School of Public Health, New Haven, CT USA

**Keywords:** Randomized controlled trials, Correlation structures, Malaria, Robust variance estimation, Mixed-effects regression

## Abstract

**Background:**

The effectiveness of malaria vector control interventions is often evaluated using cluster randomized trials (CRT) with outcomes assessed using repeated cross-sectional surveys. A key requirement for appropriate design and analysis of longitudinal CRTs is accounting for the intra-cluster correlation coefficient (ICC). In addition to exchangeable correlation (constant ICC over time), correlation structures proposed for longitudinal CRT are block exchangeable (allows a different within- and between-period ICC) and exponential decay (allows between-period ICC to decay exponentially). More flexible correlation structures are available in statistical software packages and, although not formally proposed for longitudinal CRTs, may offer some advantages. Our objectives were to empirically explore the impact of these correlation structures on treatment effect inferences, identify gaps in the methodological literature, and make practical recommendations.

**Methods:**

We obtained data from a parallel-arm CRT conducted in Tanzania to compare four different types of insecticide-treated bed-nets. Malaria prevalence was assessed in cross-sectional surveys of 45 households in each of 84 villages at baseline, 12-, 18- and 24-months post-randomization. We re-analyzed the data using mixed-effects logistic regression according to a prespecified analysis plan but under five different correlation structures as well as a robust variance estimator under exchangeable correlation and compared the estimated correlations and treatment effects. A proof-of-concept simulation was conducted to explore general conclusions.

**Results:**

The estimated correlation structures varied substantially across different models. The unstructured model was the best-fitting model based on information criteria. Although point estimates and confidence intervals for the treatment effect were similar, allowing for more flexible correlation structures led to different conclusions based on statistical significance. Use of robust variance estimators generally led to wider confidence intervals. Simulation results showed that under-specification can lead to coverage probabilities much lower than nominal levels, but over-specification is more likely to maintain nominal coverage.

**Conclusion:**

More flexible correlation structures should not be ruled out in longitudinal CRTs. This may be particularly important in malaria trials where outcomes may fluctuate over time. In the absence of robust methods for selecting the best-fitting correlation structure, researchers should examine sensitivity of results to different assumptions about the ICC and consider robust variance estimators.

**Supplementary Information:**

The online version contains supplementary material available at 10.1186/s12874-023-01871-2.

## Introduction

Cluster randomized trials (CRTs) are commonly used to evaluate the effectiveness of different types of insecticide-treated nets against malaria, a parasitic disease transmitted by mosquito vectors [1]. In these trials, randomization often involves entire communities, villages or larger administrative units [2], referred to as “clusters”. Cluster randomization can simplify logistics and help avoid contamination among individuals allocated to different types of nets within the same geographical area. Despite its advantages in this setting, cluster randomization complicates trial design and analysis due to the necessity to account for the similarity of responses from multiple individuals in the same cluster. This similarity is usually measured using the intra-cluster correlation coefficient (ICC) [3]. A typical assumption in CRTs is that the correlation between any two individuals in the same cluster is constant, called an “exchangeable correlation” [4]. When a CRT is analyzed using mixed-effects regression, an exchangeable correlation is obtained by including a random intercept for clusters, in which case the ICC can be estimated as the ratio of the between-cluster variance to the total variance of the outcome.

In malaria trials, outcomes (e.g., prevalence of malaria infection) are often assessed using repeated cross-sectional surveys before and after introduction of the intervention. This design is known as a longitudinal CRT. Longitudinal CRTs are more complex to design and analyze. Substantial development for novel types of longitudinal CRT designs such as stepped wedge and multiple cluster cross-over designs has taken place in recent years [4–6]. A focus of this literature has been on accounting for complex within- and between-period intra-cluster correlations. In short, when data across all time periods are analyzed in one overall model, the assumption of an exchangeable ICC may not be plausible: [5–7] it is more reasonable to allow the correlation between two individuals measured in the same cluster but in different periods (called the between-period ICC) to be different (often weaker) than the correlation between two individuals measured in the same cluster but in the same period (called the within-period ICC) [5, 6, 8]. Two types of correlation structures that allow both a within-period and between-period ICC are nested exchangeable [5, 7] and exponential decay [6]. Sample size calculation procedures for longitudinal CRTs under exchangeable, nested exchangeable and exponential decay structures are now readily available [9]. Parameters that need to be specified for sample size calculation include the within-period ICC and the Cluster Autocorrelation Coefficient (CAC), defined as the ratio of the between-period ICC to within-period ICC, or alternatively, as the rate of decay per period.

Despite substantial methodological development for novel longitudinal designs in recent years, relatively less attention has been paid to longitudinal parallel-arm CRTs. Parallel-arm CRTs differ from stepped-wedge and cluster cross-over designs in some important ways. For example, when the treatment effect is expected to vary over time, the analysis of post-intervention data from a parallel-arm design may involve data pooled across all periods with treatment, period and treatment-by-period interactions specified as fixed effects and adjusting for the baseline measure (e.g., cluster malaria prevalence before randomization) as a covariate. Period-specific treatment effect estimates may then be obtained as least square mean differences from the model. In contrast, existing methods for SW-CRTs and cluster cross-over designs typically assume that the intervention effect of interest is the time-averaged difference between intervention and control conditions. Furthermore, cluster cross-over and stepped wedge CRTs are usually designed with equal step lengths, whereas parallel-arm CRTs may have different durations between successive cross-sections, which means that some types of correlation structures (e.g., exponential decay) may not be meaningful. Finally, more flexible correlation structures may be required in malaria trials where seasonal effects, and complex time by treatment interactions may imply that correlations do not necessarily decay or do not decay in a consistent pattern.

In this manuscript, we use data from a parallel-arm longitudinal malaria vector control trial conducted in Tanzania [10] to introduce readers to five different types of correlation structures available for longitudinal CRTs. We present empirical estimates for different correlation structures using data from this trial and consider the implications for inferences about the treatment effect. We identify gaps in the methodological literature and make practical recommendations for investigators designing and analysing longitudinal parallel-arm CRTs. Finally, we present results from a proof-of-concept simulation study to demonstrate the potential implications of random effects misspecification.

## Correlation structures for longitudinal CRTs

A good overview of available correlation structures in linear mixed-effects regression analyses of longitudinal CRT designs is provided in Li et al. [11] They distinguish between three main correlation structures for cross-sectional designs with continuous outcomes: exchangeable, nested exchangeable, and exponential decay [4–6]. Table 1 provides diagrams corresponding to these structures. Unlike an exchangeable correlation, the nested exchangeable correlation assumes a constant between-period ICC which is allowed to differ from the within-period ICC. An exponential decay structure allows the between-period ICC to decay at an exponential rate over time. The underlying analytical models and definitions for these correlation structures under linear mixed models are available in Li et al. [11] For binary outcomes, similar correlation structures can be defined on the latent outcome scale by applying the logistic variance definition ($\frac{\pi^2}{3}$) in place of the usual residual variance [12].

**Table 1:** Visualization of five possible correlation structures for longitudinal cluster randomized trials

Correlation structure	Intracluster correlation matrices
**Exchangeable**	
**Nested Exchangeable**	
**Exponential decay**	
**Toeplitz**	
**Unstructured**	

*ρ*_*w*_/*ρ*_*wi*_ = within-period ICC, *ρ*_*b*_/*ρ*_*bi*_ = between-period ICC, *ρ*_*ij*_ = the correlation between two within-cluster observations collected during the i-th and j-th period, *r* = cluster autocorrelation

Each correlation matrix shows a design with 3 periods and 2 cluster-period sizes. Across blocks, it shows the between period correlations between any individual from two periods. Within each block, correlations show the correlation between two individuals within the same period. *ρ*_*ij*_ is equal to *ρ*_*ji*_

Although parsimonious, these three correlation structures may be unnecessarily restrictive. Two alternative correlation structures are Toeplitz and unstructured models [13], also illustrated in Table 1. Assuming the number of clusters is relatively large, these models could feasibly be fitted using available statistical software packages, although such models have not been formally proposed for analyzing longitudinal CRTs. The Toeplitz and exponential decay structures are similar in that they assume equally spaced measurements and equal pairwise correlations between measurements that are the same distance apart, but under the Toeplitz structure, the correlations are not required to decay or decay in a consistent pattern. The unstructured structure is the most flexible allowing the pairwise correlations to be different (and even increase) at each timepoint, and it does not force the within-period ICCs to be equal. As such, the unstructured correlation does not require measurements to be equally spaced.

Kasza and Forbes [14] considered the implications of misspecification of the correlation structure for longitudinal CRTs, specifically assuming an exchangeable or nested exchangeable correlation when exponential decay holds. They showed that standard errors can be either over- or under-estimated, depending on the type of CRT design: stepped wedge, cross-over or parallel with post-baseline repeated measures. The choice of correlation structure therefore has important implications for the inferences from the trial, but to date, there is no definitive method for determining the best correlation structure for longitudinal CRTs [15]. For continuous outcomes, one possibility is to use information criteria, such as Akaike Information Criteria (AIC) and Bayesian Information Criteria (BIC). Although Murray et al. [16] pointed out that the AIC- and BIC-preferred models do not always protect the Type I error rate, a recent publication by Rezaei-Darzi et al. [17] found that for continuous outcomes with larger numbers of clusters, periods and participants per cluster-period, AIC and BIC both perform adequately in identifying the correct within-cluster correlation structure. However, for smaller total sample sizes, these criteria do not perform well and when the degree of dependence between observations in adjacent periods is large and the number of periods is small, neither criterion is able to distinguish between different correlation structures. These authors recommend that, if sample sizes are adequate, AIC or BIC can be used in the absence of other compelling justifications for a specific correlation structure and that researchers conduct sensitivity analyses under alternative correlation structures. Other studies have investigated using AIC and BIC for various conditions, including covariance heterogeneity, and found that they performed rather poorly [18]. To our knowledge, the use of these information criteria has not been examined for non-continuous outcomes. In the absence of reliable methods for choosing the best-fitting correlation structure, an alternative is to use robust variance estimators (RVE) which can yield consistent variance estimates for the treatment effect even when the correlation is misspecified [19].

## Motivating example

The motiving example is a longitudinal, parallel-arm CRT conducted in Northwestern Tanzania between October 2018 and January 2021 [10, 20]. This trial included 84 clusters comprised of 72 villages covering a total of 39,307 households. The trial aimed to determine the comparative effectiveness of three new dual active ingredient long-lasting insecticidal nets (LLINs) that combine a pyrethroid insecticide with another insecticide or synergist in reducing malaria transmitted by mosquito vectors that are resistant to standard insecticides. The three interventions, all different types of dual active ingredient LLINs (denoted hereafter as intervention 2, 3 and 4), were compared against a single-insecticide, pyrethroid-only LLIN (intervention 1).

Malaria prevalence in children aged 6 months to 14 years (the primary trial outcome) was assessed through a baseline survey in 2018, and trial clusters were subsequently allocated to one of the four intervention arms by covariate constrained randomization with 21 clusters allocated to each arm. To evaluate the impact of trial interventions on malaria prevalence, repeated cross-sectional measures of malaria prevalence were then taken at fixed (equally spaced) timepoints: 12-, 18- and 24-months following the distribution of LLINs in the study area in January 2019. The primary endpoint was specified as malaria prevalence at 24 months. The baseline survey and each subsequent cross-sectional measure targeted 50 children per cluster. The average malaria prevalence at baseline ranged from 42.0%–46.6% across intervention arms. The overall prevalence of malaria at each post-intervention timepoint was 22.0%, 46.7% and 37.3%. Malaria prevalence was higher in all arms at the 18-month timepoint, which occurred following the main malaria transmission season in July/August of 2021, compared to the 12- and 24-month timepoints that occurred following the short malaria transmission seasons in January/February of 2020 and 2021, respectively. In this trial, according to a prespecified analytical plan, all three post-intervention measurements were analyzed in a single model with fixed effects for intervention, period, period-by-intervention, logit-transformed baseline cluster mean malaria prevalence, and all variables used in the restricted randomization. The correlation structure was specified as nested exchangeable. Further details on the trial design and the results of the primary trial analysis are available in the published protocol [20] and primary trial report [10].

## Methods of analysis

We re-analyzed the trial data using mixed-effects logistic regression following the same analytical plan as in the published trial but fitting five different correlation structures: (1) exchangeable, (2) nested exchangeable, (3) exponential decay, (4) Toeplitz, and (5) unstructured. In addition, we fitted the models using the RVE under an exchangeable working correlation. Estimates of intervention effects on malaria prevalence at each post-intervention timepoint (i.e., 12-, 18- and 24-months) were obtained as adjusted and unadjusted least square mean differences from the model. These results were reported as odds ratios (ORs) with associated 95% confidence intervals (CIs). All analyses were conducted in SAS 9.4 (Cary, NC, USA) using PROC GLIMMIX. The SAS code for conducting the analyses and estimating the correlations is provided in the Additional file 1. The estimation procedure was adaptive quadrature [21, 22], and we used the default degree of freedom option (between-within method) in SAS. As the investigators prespecified the comparison of each new type of net to the reference net as the primary comparisons of interest [23], the alpha value for evaluating statistical significance was set as 0.017. For each assumed correlation structure, estimates for the correlations were obtained first on the logit scale [12] assuming the residual variance to be $\frac{\pi^2}{3}$, and then on the proportions scale using linear mixed model approximations. We present both covariate-adjusted and unadjusted ICCs. We also extracted information criteria, corrected AIC (AICC), BIC, and negative log-likelihood from each model.

## Results

### Estimated correlations

The estimated correlation structures on the logit scale are presented in Table 2. (The estimates on the proportions scale are presented in Additional file 2, Table S1). Not surprisingly, the exchangeable model (which assumes a constant ICC across all periods) returned the lowest ICC (0.16 before covariate adjustment). After covariate adjustment, the ICC decreased substantially to 0.05, which indicates that the covariates explain a substantial proportion of the between-cluster variability in this trial. As expected, under structures which allowed for a different between-period ICC, the within-period ICC was higher (0.19 under nested exchangeable, exponential decay and Toeplitz structures before covariate adjustment; 0.08 with covariate adjustment). The estimated correlation matrix was similar between exponential decay and Toeplitz models, which indicates the extra flexibility of the Toeplitz model may not be necessary. Most notably, the estimated within-period ICC in the unstructured correlation varied over time from 0.091 in the first period, 0.052 in the middle period, to 0.118 in the final period. All other correlation structures forced the within-period ICC to be the same over time. Furthermore, in the unstructured correlation, the between-period ICC did not decay every period but increased from the second to third period.

**Table 2:** Estimated ICC values under five different correlation structures for the example trial on the logit scale, unadjusted and adjusted for prespecified covariates

	Unadjusted			Adjusted		
Correlation structures	WPICC (***ρ***_***w***_)	CAC	Intra-cluster correlation matrix^a^	WPICC (***ρ***_***w***_)	CAC	Intra-cluster correlation matrix^a^
**Exchangeable**	0.160	1	$$\left(\begin{array}{ccc}0.160& 0.160& 0.160\\ {}0.160& 0.160& 0.160\\ {}0.160& 0.160& 0.160\end{array}\right)$$	0.054	1	$$\left(\begin{array}{ccc}0.054& 0.054& 0.054\\ {}0.054& 0.054& 0.054\\ {}0.054& 0.054& 0.054\end{array}\right)$$
**Nested Exchangeable**	0.187	0.774	$$\left(\begin{array}{ccc}0.187& 0.145& 0.145\\ {}0.145& 0.187& 0.145\\ {}0.145& 0.145& 0.187\end{array}\right)$$	0.084	0.421	$$\left(\begin{array}{ccc}0.084& 0.035& 0.035\\ {}0.035& 0.084& 0.035\\ {}0.035& 0.035& 0.084\end{array}\right)$$
**Exponential decay**	0.189	0.822	$$\left(\begin{array}{ccc}0.189& 0.156& 0.128\\ {}0.156& 0.189& 0.156\\ {}0.128& 0.156& 0.189\end{array}\right)$$	0.084	0.505	$$\left(\begin{array}{ccc}0.084& 0.043& 0.021\\ {}0.043& 0.084& 0.043\\ {}0.021& 0.043& 0.084\end{array}\right)$$
**Toeplitz**	0.189	–	$$\left(\begin{array}{ccc}0.189& 0.153& 0.136\\ {}0.153& 0.189& 0.153\\ {}0.136& 0.153& 0.189\end{array}\right)$$	0.084	–	$$\left(\begin{array}{ccc}0.084& 0.041& 0.028\\ {}0.041& 0.084& 0.041\\ {}0.028& 0.041& 0.084\end{array}\right)$$
**Unstructured**	–	–	$$\left(\begin{array}{ccc}0.216& 0.140& 0.149\\ {}0.140& 0.156& 0.150\\ {}0.149& 0.150& 0.199\end{array}\right)$$	–	–	$$\left(\begin{array}{ccc}0.091& 0.017& 0.039\\ {}0.017& 0.052& 0.054\\ {}0.039& 0.054& 0.118\end{array}\right)$$

WPICC: Within-period ICC, CAC: Cluster autocorrelation coefficient

^a^Each cell [i, j] represent correlation between two within-cluster individuals collected in different i and j periods

Information criteria under the different models are reported in Table 3. There was some disagreement between the criteria: AICC reached its minimum value for the unstructured model whereas BIC (which is known to prefer a simpler model) indicated that exponential decay model resulted in the best-fitting model.

**Table 3:** Fit statistics for the example trial, using mixed-effects logistic regression models under five different correlation structures, adjusting for pre-specified covariates (minimized values bolded)

Model	AICC	BIC	-2 Log Likelihood
**Exchangeable**	16,432.99	16,479.13	16,394.94
**Nested Exchangeable**	16,299.18	16,347.73	16,259.12
**Exponential decay**	16,298.42	**16,346.98**	16,258.36
**Toeplitz**	16,299.72	16,350.70	16,257.66
**Unstructured**	**16,291.87**	16,350.12	**16,243.78**

### Estimated treatment effects from models

**Fig. 1:**
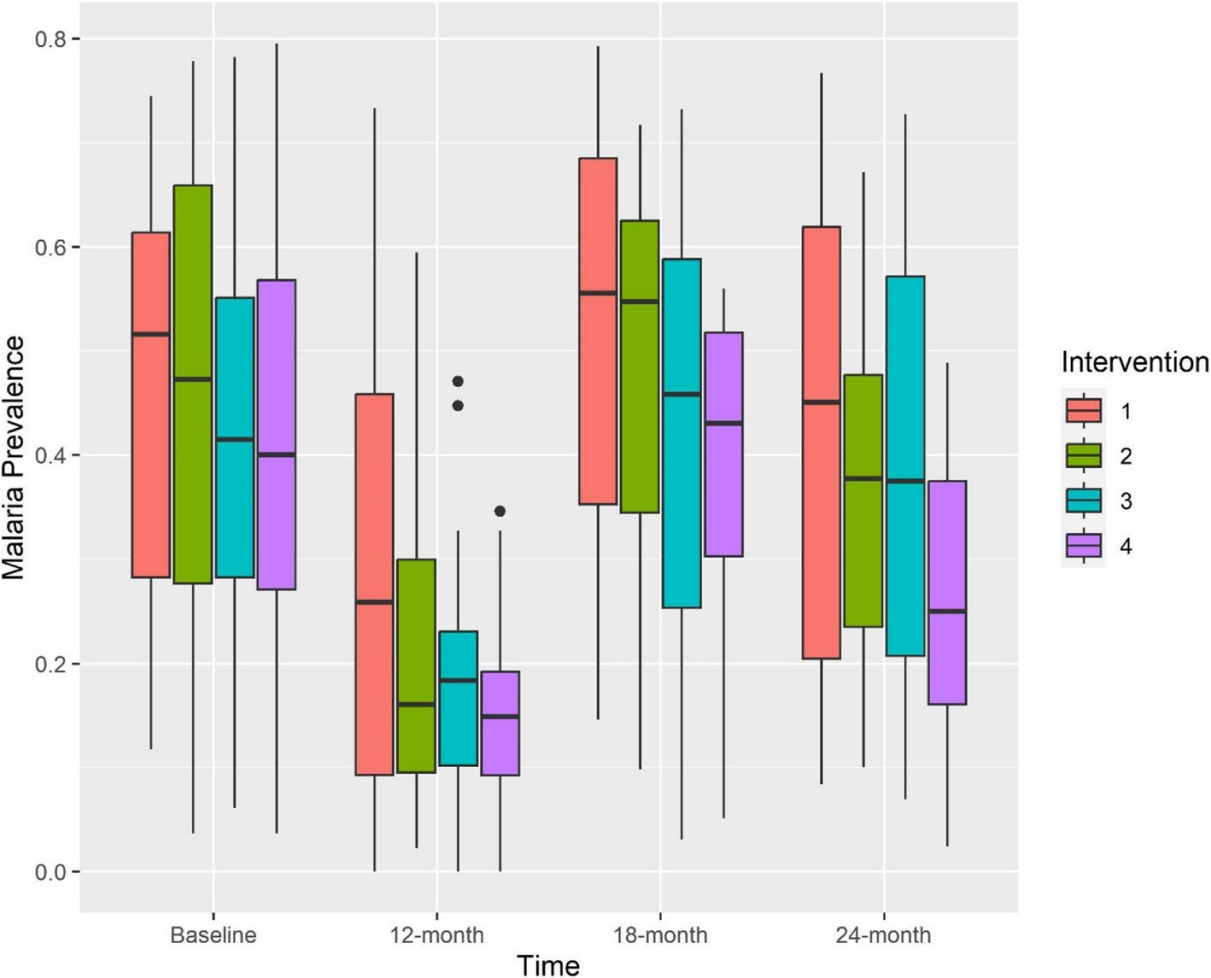
Boxplot of cluster-specific malaria prevalence at baseline, 12 months, 18 months and 24 months by intervention

**Fig. 2:**
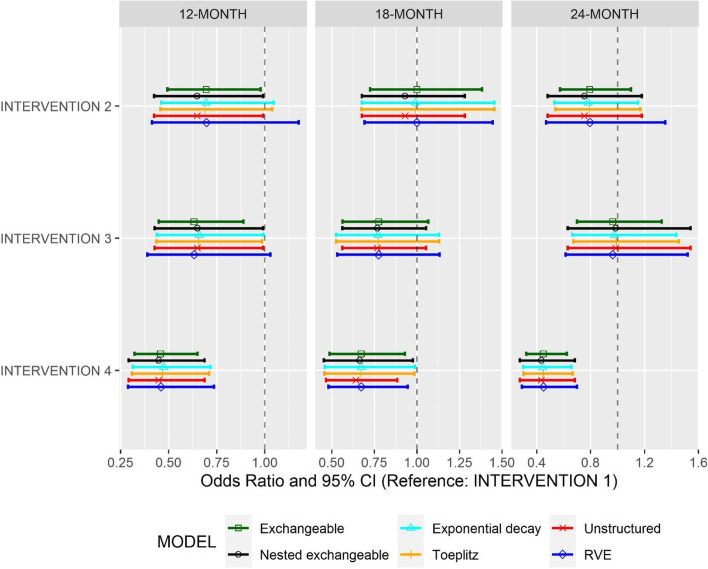
Comparison of intervention effect estimates and 95% confidence intervals using mixed-effects logistic regression adjusted for prespecified covariates and assuming five different correlation structures as well as assuming robust variance estimators with exchangeable correlation

The observed cluster-specific malaria prevalence estimates in each arm at baseline, 12, 18 and 24 months are presented in Fig. 1 using boxplots. The model-based least square mean differences, expressed as adjusted ORs with 95% CIs, obtained from the covariate-adjusted models at each timepoint are presented in Fig. 2 (the unadjusted estimates are presented in Additional file 2, Fig. S1). For all intervention arms, the exchangeable model generally resulted in the narrowest confidence intervals at 12- and 24 months, but unstructured had the narrowest confidence intervals at 18 months. Exponential decay and Toeplitz models often returned very similar ORs and confidence intervals. It is worth noting that treatment effect estimates showed an “up and down” trend over time with intervention effects generally being stronger at 12 and 24 months. This is because the 18-month point was following the long rainy season where the prevalence of malaria was higher and more variable in all arms (see Fig. 1). Intervention 4 was the only type of net with confidence intervals excluding the null at all time points; interventions 2 and 3 had confidence intervals overlapping with the null in at least some of the correlation structures at all time points. Unsurprisingly, use of the RVE led to estimates similar to those from the exchangeable models but with wider confidence intervals. All adjusted and unadjusted estimates and their confidence intervals are presented in the Additional file 2, Table S2-S3.

## Discussion

Correct specification of the correlation structure is important in the design and analysis of longitudinal CRTs. A mis-specified correlation structure could reduce statistical efficiency or worse, lead to incorrect inferences. Although models that allow correlations to decay have received substantial attention in the literature for stepped wedge and cluster cross-over designs, more flexible correlation structures that do not require a decay, may be warranted in the case of longitudinal parallel-arm CRTs, especially when intervals are unequally spaced. In this re-analysis of a previously published longitudinal, parallel-arm CRT to evaluate malaria vector control interventions, we empirically compared the implications of alternative assumptions about the correlation structures on inferences about the treatment effect.

First, we demonstrated that within-period ICCs can be substantially different over time — yet correlation structures currently recommended in the methodological literature (i.e., nested exchangeable and exponential decay) assume a common within-period ICC over time. Second, we demonstrated that between-period ICCs do not necessarily decay. For both reasons, more flexible correlation structures (e.g., unstructured) may be required for parallel-arm longitudinal CRTs. This flexibility seems to be important in general for malaria trials. The heterogeneity in malaria transmission in space and time is likely to change between-cluster variations, thus requiring more complex correlation structures. For example, due to seasonal effects in the prevalence of malaria, it is possible that the between-cluster variability changes over time, and due to complex mechanisms of action for the intervention (e.g., an initial strong effect followed by a rebound), the correlation between different measurements from the same cluster may increase rather than decrease with increasing time separation. More generally, variation in within-period correlations could also arise due to limitations in the use of correlations to quantify similarity for binary data with quite varying prevalence.

In our analyses, although one might expect more efficient estimation of the treatment effect under an exchangeable model (due to a smaller number of parameters to be estimated), we found that the confidence interval around the estimated treatment effect was narrower under the unstructured model at the 18-month timepoint. This may be related to the fact that the within-period ICC at 18 months was less than half the within-period ICC at 24 months, and only the unstructured model would allow for this flexibility. Our results under a Bonferroni correction showed that different conclusions would have been reached under different correlation structures when using only statistical significance (Additional file 2, Table S2).

Our analysis demonstrates that there are some gaps in the existing methodological literature. Information criteria may not perform reliably in choosing between alternative models and further work is required to determine best strategies for model selection. A potential strategy is to always favour a more flexible correlation structure, i.e., to over-specify the correlation structures. Kasza and Forbes [14] found that for continuous outcomes, over-specification asymptotically does not lead to bias. However, this approach may not always be feasible. A more complex correlation structure requires more parameters to be estimated, which requires larger cluster sizes and more clusters. Therefore, although complex correlation structures appear to be more attractive, it is not always practical, especially with small CRTs. Furthermore, computational challenges may be encountered when fitting models with more complex correlation structures. Some mitigating strategies may be available [24], for example, in SAS, METHOD = FASTQUAD may offer a less computationally expensive solution [22, 25]. One practical strategy to investigate whether a more complex correlation structure is essential may be to empirically examine the assumption of common within-period ICCs by fitting an initial unstructured model or fitting separate models at each cross-section of time; if the estimated ICCs vary considerably over time, models which assume a common within-period ICC over time may not be reasonable.

An alternative approach is to fit models with RVEs, which has been shown to yield consistent variance estimates even under model misspecification. However, this method generally results in larger confidence intervals than the “correctly” specified model and could lead to different conclusions about effectiveness. The loss of efficiency can be an important consideration in smaller CRTs. Ultimately, in the absence of guidelines to select the best correlation structure in longitudinal CRTs, a good recommendation is to always examine sensitivity of results to different assumptions.

## Proof-of-concept simulation

To complement our example, we conducted a proof-of-concept simulation study. We simulated data for a continuous outcome matching the malaria trial characteristics and investigated: 1) the impact of analyzing the data using exchangeable, nested exchangeable, exponential decay, and Toeplitz structures when the data were generated from unstructured models (under-specification); and 2) the impact of analyzing the data using exponential decay, Toeplitz, and unstructured models when the data were generated from exchangeable models (over-specification). For reference, the true correlation structure was also fitted. For completeness, we also examined use of RVE with an exchangeable correlation. Based on the malaria trial, the simulation study considered 84 clusters, 4 periods (one baseline and three follow-ups), a cluster period size of 45, and a treatment effect size of zero. We investigated ICCs of 0.05 and 0.1 when the true model was exchangeable. We investigated scenarios in which the within-period ICC increases linearly, decreases linearly, or changes in any direction when the true model was unstructured. We considered the implications for bias of the estimated treatment effect and coverage of a 95% confidence interval (CI) around the estimated treatment effect; we also examined the relative error of the model-based standard error (SE) to the Monte Carlo standard deviation, and the ratio of the SE from the model under consideration to that under the true data generating model. The details for the simulation scenarios and corresponding results are presented in the Additional file 3 and are summarized here.

Although random effects were mis-specified, estimated treatment effects were unbiased in all scenarios examined. When the model was under-specified, we found that the bias of the model-based standard error was as large as 75% under the exchangeable model, leading to 95% coverage around 40%; when we allowed for a decay, coverage was closer to, but still below, the nominal level. Depending on how the within-period and between-period ICCs changed over time, the exponential decay and Toeplitz models achieved coverage either below or above 95%, and the estimated model-based SE were either smaller or larger than the model-based SE from the unstructured model. Adding RVEs to the exchangeable model maintained the validity of statistical inferences while a loss in efficiency was noted (the SE was inflated by 7% to 49%).

When the model was over-specified, we found that the nominal level of coverage was maintained. The average model-based standard error was slightly inflated (by no more than 7%), indicating a potential loss of efficiency. It is worthwhile noting that over-specification led to substantial non-convergence: among all simulation runs, more than 86% of Toeplitz and unstructured models failed to converge. In this case, the exchangeable model with RVE was a good solution with minimal loss of efficiency from the use of RVE.

To summarize, our limited simulation study suggests that fitting more flexible correlation structures may be required to maintain statistical validity; in cases of non-convergence, the exchangeable model with RVEs is a reasonable solution, although the loss of efficiency can be large in some cases.

## Conclusions and recommendations for future research

We have shown that less restrictive correlation structures, such as Toeplitz and unstructured, may be more reasonable for some longitudinal CRTs. However, their properties have not been well-studied in CRTs, and we are unaware of any sample size calculation methodologies for these two correlation structures. Future papers and more extensive simulation studies may examine implications of choosing exchangeable, nested exchangeable or exponential decay models, when a more flexible correlation structure holds. Thompson et al. [26] have shown that mis-specifying random effects could lead to serious under coverage and biased treatment estimates for mixed-effects logistic regression analysis of binary outcomes. However, they only considered simple correlation structures (e.g., exchangeable, and nested exchangeable). Our limited simulation study showed that similar results may apply in the case of more complex correlation structures, but future work is required to specifically address the implications of misspecification under more complex correlation structures. Investigators also urgently need guidance as to selecting the most appropriate correlation structures, especially for binary outcomes.

In this paper we assumed that the analytical model is based on data from all periods with a treatment-by-period interaction and with least square mean differences obtained from the model to express the intervention effect. An alternative analytical strategy which avoids the added complexity of the longitudinal trial is to analyze data from each timepoint separately. Planned future research will examine how to choose a relevant estimand for parallel-arm longitudinal CRTs as well as the advantages and disadvantages of analyzing separate cross-sections as opposed to data from all periods in one overall analytical model.

RVEs have been widely used in generalized estimating equations (GEEs) but not often in the context of mixed-effect models. In principle, while RVEs are robust against misspecification of the correlation structure, they might yield more efficient variance estimates when the specified correlation structure is closer to the true model. An important question to address is what working correlation structure should be specified to obtain the largest gain in efficiency without unnecessarily complicating the model. More comprehensive simulation studies are required to quantify the actual gain of using RVEs with mixed-effects regression in longitudinal CRTs as compared to model-based variance estimators when the working correlation structure is only mildly different from the true correlation structure.

The main contribution of this paper is to provide a case study where more flexible correlation structures are desirable. Although Toeplitz and unstructured correlation structures are commonly considered in individually randomized longitudinal trials, to our knowledge, they have not been formally considered in longitudinal CRTs. Further theoretical and more extensive simulation work is required to generate firm recommendations for use of these methods in practice. This study can serve as a motivation for future theoretical exploration of more complex correlation structures in longitudinal parallel-arm CRTs.

## Supplementary Information

**Additional file 1.**

**Additional file 2.**

**Additional file 3.**

## Data Availability

The datasets analysed during the current study are not publicly available. Deidentified data and data dictionary will be made available at the end of the third year of trial follow-up upon reasonable request to the corresponding author of the original trial.

## Abbreviations

ICC: Intra-cluster correlation coefficient
BPICC: Between-period ICC
CAC: Cluster autocorrelation coefficient
CRT: Cluster randomized trial
GEE: Generalized estimating equations
LLINs: Long-lasting insecticidal nets
OR: Odds ratio
RVE: Robust variance estimation
WPICC: Within-period ICC
