# Supplementary material for: Accounting for complex intracluster correlations in longitudinal cluster randomized trials: a case study in malaria vector control
Source: BMC Med Res Methodol. 2023 Mar 17;23:64. doi: 10.1186/s12874-023-01871-2 (PMC10021932; doi:10.1186/s12874-023-01871-2)
Supplement: Supplementary file 1 — Additional file 1. [file 12874_2023_1871_MOESM1_ESM.docx]

**Additional file 1: SAS code to fit models with five correlation structures**

SAS code

/*PROJECT: Accounting for complex intracluster correlations in longitudinal cluster randomized

trials: a case study in malaria vector control

Supplementary code: how to fit data with various correlation structures*/

/*LOAD DATASET: */

***proc*** **import** *out***=** hsb2 datafile **=** "Path to the dataset"**;**

***run*;**

/*unstructured correlation structure*/

***PROC*** **GLIMMIX** ***DATA*=**hsb2 NOCLPRINT METHOD **=** QUAD**;**

CLASS clusterno surveyno intervention_n **(***REF***=**"1"**);**

**MODEL** rdtresults **(**EVENT**='1')=** intervention_n surveyno intervention_n*****surveyno rdtpos_log itnuse_log poor_log pop_100 fun_log gam_log

**/** DIST**=**BINARY LINK**=**LOGIT SOLUTION**;**

RANDOM surveyno **/** SUBJECT**=**clusterno TYPE**=**UN G**;**

LSMEANS intervention_n*****surveyno**/**SLICE**=**surveyno DIFF CL ODDSRATIO**;**

ods output Diffs**=**UNlsmeans covparms**=**UNcovparms fitstatistics**=**UNfitstatistics solutionf**=**UNsolutionf**;**

***RUN*;**

/*Topelitz correlation structure*/

***PROC*** **GLIMMIX** ***DATA*=**hsb2 NOCLPRINT METHOD **=** QUAD**;**

CLASS clusterno surveyno intervention_n **(***REF***=**"1"**);**

**MODEL** rdtresults **(**EVENT**='1')=** intervention_n surveyno intervention_n*****surveyno rdtpos_log itnuse_log poor_log pop_100 fun_log gam_log

**/** DIST**=**BINARY LINK**=**LOGIT SOLUTION**;**

RANDOM surveyno **/** SUBJECT**=**clusterno TYPE**=**TOEP G **;**

LSMEANS intervention_n*****surveyno**/**SLICE**=**surveyno DIFF CL ODDSRATIO**;**

ods output Diffs**=**TPlsmeans covparms**=**TPcovparms fitstatistics**=**TPfitstatistics solutionf**=**TPsolutionf**;**

***RUN*;**

/*Exponential decay correlation structure*/

***PROC*** **GLIMMIX** ***DATA*=**hsb2 NOCLPRINT METHOD **=** QUAD**;**

CLASS clusterno surveyno intervention_n **(***REF***=**"1"**);**

**MODEL** rdtresults **(**EVENT**='1')=** intervention_n surveyno intervention_n*****surveyno rdtpos_log itnuse_log poor_log pop_100 fun_log gam_log

**/** DIST**=**BINARY LINK**=**LOGIT SOLUTION**;**

RANDOM surveyno **/** SUBJECT**=**clusterno TYPE**=**AR**(1)** **;**

LSMEANS intervention_n*****surveyno**/**SLICE**=**surveyno DIFF CL ODDSRATIO**;**

ods output Diffs**=**ARlsmeans covparms**=**ARcovparms fitstatistics**=**ARfitstatistics solutionf**=**ARsolutionf**;**

***RUN*;**

/*Nested exchangeable*/

***PROC*** **GLIMMIX** ***DATA*=**hsb2 NOCLPRINT METHOD **=** QUAD**;**

CLASS clusterno surveyno intervention_n **(***REF***=**"1"**);**

**MODEL** rdtresults **(**EVENT**='1')=** intervention_n surveyno intervention_n*****surveyno rdtpos_log itnuse_log poor_log pop_100 fun_log gam_log

**/** DIST**=**BINARY LINK**=**LOGIT SOLUTION**;**

RANDOM intercept surveyno **/**SUBJECT**=**clusterno TYPE**=**VC**;**

** RANDOM clusterno clusterno*surveyno;

LSMEANS intervention_n*****surveyno**/**SLICE**=**surveyno DIFF CL ODDSRATIO**;**

ods output Diffs**=**NElsmeans covparms**=**NEcovparms fitstatistics**=**NEfitstatistics solutionf**=**NEsolutionf**;**

***RUN*;**

/*Exchangeable*/

***PROC*** **GLIMMIX** ***DATA*=**hsb2 NOCLPRINT METHOD **=** QUAD**;**

CLASS clusterno surveyno intervention_n **(***REF***=**"1"**);**

**MODEL** rdtresults **(**EVENT**='1')=** intervention_n surveyno intervention_n*****surveyno rdtpos_log itnuse_log poor_log pop_100 fun_log gam_log

**/**covb DIST**=**BINARY LINK**=**LOGIT SOLUTION**;**

RANDOM intercept**/**SUBJECT**=**clusterno**;**

LSMEANS intervention_n*****surveyno**/**SLICE**=**surveyno DIFF CL ODDSRATIO**;**

ods output Diffs**=**HHlsmeans covparms**=**HHcovparms fitstatistics**=**HHfitstatistics solutionf**=**HHsolutionf**;**

***RUN*;**

/*Save output: Save least square means in excel*/

ods excel file**=**"Path to save\result_adjust.xlsx" options**(**sheet_name**=**"UN: LS"**);**

***proc*** **print** ***data*=**work**.**UNlsmeans**;**

title "LS Difference UN"**;**

***run*;**

ods excel options**(**sheet_name**=**"TP: LS"**);**

***proc*** **print** ***data*=**work**.**TPlsmeans**;**

title "LS Difference TP"**;**

***run*;**

ods excel options**(**sheet_name**=**"AR: LS"**);**

***proc*** **print** ***data*=**work**.**ARlsmeans**;**

title "LS Difference AR"**;**

***run*;**

ods excel options**(**sheet_name**=**"NE: LS"**);**

***proc*** **print** ***data*=**work**.**NElsmeans**;**

title "LS Difference NE"**;**

***run*;**

ods excel options**(**sheet_name**=**"HH: LS"**);**

***proc*** **print** ***data*=**work**.**HHlsmeans**;**

title "LS Difference HH"**;**

***run*;**

ods excel close**;**

/*Save output: Save variance component in excel*/

ods excel file**=**"vcov_logit.xlsx" options**(**sheet_name**=**"H+H: fitstat"**);**

***proc*** **print** ***data*=**work**.**HHfitstatistics**;**

title "Fit statistics"**;**

***run*;**

ods excel options**(**sheet_name**=**"H+H: solutionf"**);**

***proc*** **print** ***data*=**work**.**HHsolutionf**;**

title "Fixed effects"**;**

***run*;**

ods excel options**(**sheet_name**=**"H+H: covparms"**);**

***proc*** **print** ***data*=**work**.**HHcovparms**;**

title "Covariance parameters"**;**

***run*;**

ods excel options**(**sheet_name**=**"H-G: fitstat"**);**

***proc*** **print** ***data*=**work**.**NEfitstatistics**;**

title "Fit statistics"**;**

***run*;**

ods excel options**(**sheet_name**=**"H-G: solutionf"**);**

***proc*** **print** ***data*=**work**.**NEsolutionf**;**

title "Fixed effects"**;**

***run*;**

ods excel options**(**sheet_name**=**"H-G: covparms"**);**

***proc*** **print** ***data*=**work**.**NEcovparms**;**

title "Covariance parameters"**;**

***run*;**

ods excel options**(**sheet_name**=**"ED: fitstat"**);**

***proc*** **print** ***data*=**work**.**ARfitstatistics**;**

title "Fit statistics"**;**

***run*;**

ods excel options**(**sheet_name**=**"ED: solutionf"**);**

***proc*** **print** ***data*=**work**.**ARsolutionf**;**

title "Fixed effects"**;**

***run*;**

ods excel options**(**sheet_name**=**"ED: covparms"**);**

***proc*** **print** ***data*=**work**.**ARcovparms**;**

title "Covariance parameters"**;**

***run*;**

ods excel options**(**sheet_name**=**"UN: fitstat"**);**

***proc*** **print** ***data*=**work**.**UNfitstatistics**;**

title "Fit statistics"**;**

***run*;**

ods excel options**(**sheet_name**=**"UN: solutionf"**);**

***proc*** **print** ***data*=**work**.**UNsolutionf**;**

title "Fixed effects"**;**

***run*;**

ods excel options**(**sheet_name**=**"UN: covparms"**);**

***proc*** **print** ***data*=**work**.**UNcovparms**;**

title "Covariance parameters"**;**

***run*;**

ods excel options**(**sheet_name**=**"TP: fitstat"**);**

***proc*** **print** ***data*=**work**.**TPfitstatistics**;**

title "Fit statistics"**;**

***run*;**

ods excel options**(**sheet_name**=**"TP: solutionf"**);**

***proc*** **print** ***data*=**work**.**TPsolutionf**;**

title "Fixed effects"**;**

***run*;**

ods excel options**(**sheet_name**=**"TP: covparms"**);**

***proc*** **print** ***data*=**work**.**TPcovparms**;**

title "Covariance parameters"**;**

***run*;**

ods excel close**;**

/*Model with robust variance estimator*/

***PROC*** **GLIMMIX** ***DATA*=**hsb2 NOCLPRINT EMPIRICAL**=**FIRORES**;**

CLASS clusterno surveyno intervention_n **(***REF***=**"1"**);**

**MODEL** rdtresults **(**EVENT**='1')=** intervention_n surveyno intervention_n*****surveyno

**/** DIST**=**BINARY LINK**=**LOGIT SOLUTION**;**

RANDOM intercept **/**SUBJECT**=**clusterno TYPE**=**VC**;**

LSMEANS intervention_n*****surveyno**/**SLICE**=**surveyno DIFF CL ODDSRATIO**;**

ods output Diffs**=**UNlsmeans covparms**=**UNcovparms fitstatistics**=**UNfitstatistics solutionf**=**UNsolutionf**;**

***RUN*;**

ods excel file**=**"Path to save\robust_unadjusted.xlsx" options**(**sheet_name**=**"UN: LS"**);**

***proc*** **print** ***data*=**work**.**UNlsmeans**;**

title "LS Difference UN"**;**

***run*;**

ods excel close**;**

/**proportion scale ICC*/

***PROC*** **MIXED** ***DATA*=**hsb2 **;**

CLASS clusterno surveyno intervention_n **(***REF***=**"1"**);**

**MODEL** rdtresults **=** intervention_n surveyno intervention_n*****surveyno rdtpos_log itnuse_log poor_log pop_100 fun_log gam_log **/** SOLUTION**;**

RANDOM surveyno **/** SUBJECT**=**clusterno TYPE**=**UN G**;**

LSMEANS intervention_n*****surveyno**/**SLICE**=**surveyno DIFF CL**;**

ods output covparms**=**UNcovparms fitstatistics**=**UNfitstatistics solutionf**=**UNsolutionf**;**

***RUN*;**

***PROC*** **MIXED** ***DATA*=**hsb2 **;**

CLASS clusterno surveyno intervention_n **(***REF***=**"1"**);**

**MODEL** rdtresults **=** intervention_n surveyno intervention_n*****surveyno rdtpos_log itnuse_log poor_log pop_100 fun_log gam_log **/** SOLUTION**;**

RANDOM surveyno **/** SUBJECT**=**clusterno TYPE**=**CS **;**

LSMEANS intervention_n*****surveyno**/**SLICE**=**surveyno DIFF CL**;**

ods output covparms**=**NEcovparms fitstatistics**=**NEfitstatistics solutionf**=**NEsolutionf**;**

***RUN*;**

***PROC*** **MIXED** ***DATA*=**hsb2 **;**

CLASS clusterno surveyno intervention_n **(***REF***=**"1"**);**

**MODEL** rdtresults **=** intervention_n surveyno intervention_n*****surveyno rdtpos_log itnuse_log poor_log pop_100 fun_log gam_log **/** SOLUTION**;**

RANDOM surveyno **/** SUBJECT**=**clusterno TYPE**=**TOEP**;**

LSMEANS intervention_n*****surveyno**/**SLICE**=**surveyno DIFF CL**;**

ods output covparms**=**TPcovparms fitstatistics**=**TPfitstatistics solutionf**=**TPsolutionf**;**

***RUN*;**

***PROC*** **MIXED** ***DATA*=**hsb2 **;**

CLASS clusterno surveyno intervention_n **(***REF***=**"1"**);**

**MODEL** rdtresults **=** intervention_n surveyno intervention_n*****surveyno rdtpos_log itnuse_log poor_log pop_100 fun_log gam_log **/** SOLUTION**;**

RANDOM clusterno**;**

LSMEANS intervention_n*****surveyno**/**SLICE**=**surveyno DIFF CL**;**

ods output covparms**=**HHcovparms fitstatistics**=**HHfitstatistics solutionf**=**HHsolutionf**;**

***RUN*;**

***PROC*** **MIXED** ***DATA*=**hsb2 **;**

CLASS clusterno surveyno intervention_n **(***REF***=**"1"**);**

**MODEL** rdtresults **=** intervention_n surveyno intervention_n*****surveyno rdtpos_log itnuse_log poor_log pop_100 fun_log gam_log **/** SOLUTION**;**

RANDOM surveyno **/** SUBJECT**=**clusterno TYPE**=**AR**(1)** **;**

LSMEANS intervention_n*****surveyno**/**SLICE**=**surveyno DIFF CL**;**

ods output covparms**=**ARcovparms fitstatistics**=**ARfitstatistics solutionf**=**ARsolutionf**;**

***RUN*;**

ods excel file**=**"vcov.xlsx" options**(**sheet_name**=**"H+H: fitstat"**);**

***proc*** **print** ***data*=**work**.**HHfitstatistics**;**

title "Fit statistics"**;**

***run*;**

ods excel options**(**sheet_name**=**"H+H: solutionf"**);**

***proc*** **print** ***data*=**work**.**HHsolutionf**;**

title "Fixed effects"**;**

***run*;**

ods excel options**(**sheet_name**=**"H+H: covparms"**);**

***proc*** **print** ***data*=**work**.**HHcovparms**;**

title "Covariance parameters"**;**

***run*;**

ods excel options**(**sheet_name**=**"H-G: fitstat"**);**

***proc*** **print** ***data*=**work**.**NEfitstatistics**;**

title "Fit statistics"**;**

***run*;**

ods excel options**(**sheet_name**=**"H-G: solutionf"**);**

***proc*** **print** ***data*=**work**.**NEsolutionf**;**

title "Fixed effects"**;**

***run*;**

ods excel options**(**sheet_name**=**"H-G: covparms"**);**

***proc*** **print** ***data*=**work**.**NEcovparms**;**

title "Covariance parameters"**;**

***run*;**

ods excel options**(**sheet_name**=**"ED: fitstat"**);**

***proc*** **print** ***data*=**work**.**ARfitstatistics**;**

title "Fit statistics"**;**

***run*;**

ods excel options**(**sheet_name**=**"ED: solutionf"**);**

***proc*** **print** ***data*=**work**.**ARsolutionf**;**

title "Fixed effects"**;**

***run*;**

ods excel options**(**sheet_name**=**"ED: covparms"**);**

***proc*** **print** ***data*=**work**.**ARcovparms**;**

title "Covariance parameters"**;**

***run*;**

ods excel options**(**sheet_name**=**"UN: fitstat"**);**

***proc*** **print** ***data*=**work**.**UNfitstatistics**;**

title "Fit statistics"**;**

***run*;**

ods excel options**(**sheet_name**=**"UN: solutionf"**);**

***proc*** **print** ***data*=**work**.**UNsolutionf**;**

title "Fixed effects"**;**

***run*;**

ods excel options**(**sheet_name**=**"UN: covparms"**);**

***proc*** **print** ***data*=**work**.**UNcovparms**;**

title "Covariance parameters"**;**

***run*;**

ods excel options**(**sheet_name**=**"TP: fitstat"**);**

***proc*** **print** ***data*=**work**.**TPfitstatistics**;**

title "Fit statistics"**;**

***run*;**

ods excel options**(**sheet_name**=**"TP: solutionf"**);**

***proc*** **print** ***data*=**work**.**TPsolutionf**;**

title "Fixed effects"**;**

***run*;**

ods excel options**(**sheet_name**=**"TP: covparms"**);**

***proc*** **print** ***data*=**work**.**TPcovparms**;**

title "Covariance parameters"**;**

***run*;**

ods excel close**;**
