# Supplementary material for: Accounting for complex intracluster correlations in longitudinal cluster randomized trials: a case study in malaria vector control
Source: BMC Med Res Methodol. 2023 Mar 17;23:64. doi: 10.1186/s12874-023-01871-2 (PMC10021932; doi:10.1186/s12874-023-01871-2)
Supplement: Supplementary file 2 — Additional file 2. [file 12874_2023_1871_MOESM2_ESM.docx]

**Table S1 Estimated ICC values on proportions scale under five correlation structures – unadjusted and adjusted for prespecified covariates**

|  | **Unadjusted** | | | **Adjusted** | | |
| --- | --- | --- | --- | --- | --- | --- |
| **Correlation structures** | **WPICC (**$\boldsymbol{\rho}_{\boldsymbol{w}}$**)** | **CAC** | **Intra-cluster correlation matrix**^a^ | **WPICC (**$\boldsymbol{\rho}_{\boldsymbol{w}}$**)** | **CAC** | **Intra-cluster correlation matrix**^a^ |
| **Exchangeable** | 0.102 | 1 | $\left( \begin{matrix} 0.102 & 0.102 & 0.102 \\ 0.102 & 0.102 & 0.102 \\ 0.102 & 0.102 & 0.102 \end{matrix} \right)$ | 0.040 | 1 | $\left( \begin{matrix} 0.040 & 0.040 & 0.040 \\ 0.040 & 0.040 & 0.040 \\ 0.040 & 0.040 & 0.040 \end{matrix} \right)$ |
| **Nested Exchangeable** | 0.125 | 0.709 | $\left( \begin{matrix} 0.125 & 0.089 & 0.089 \\ 0.089 & 0.125 & 0.089 \\ 0.089 & 0.089 & 0.125 \end{matrix} \right)$ | 0.065 | 0.597 | $\left( \begin{matrix} 0.065 & 0.039 & 0.039 \\ 0.039 & 0.065 & 0.039 \\ 0.039 & 0.039 & 0.065 \end{matrix} \right)$ |
| **Exponential decay** | 0.124 | 0.751 | $\left( \begin{matrix} 0.124 & 0.093 & 0.070 \\ 0.093 & 0.124 & 0.093 \\ 0.070 & 0.093 & 0.124 \end{matrix} \right)$ | 0.066 | 0.470 | $\left( \begin{matrix} 0.066 & 0.031 & 0.015 \\ 0.031 & 0.066 & 0.031 \\ 0.015 & 0.031 & 0.066 \end{matrix} \right)$ |
| **Toeplitz** | 0.125 | - | $\left( \begin{matrix} 0.125 & 0.093 & 0.078 \\ 0.093 & 0.125 & 0.093 \\ 0.078 & 0.093 & 0.125 \end{matrix} \right)$ | 0.066 | - | $\left( \begin{matrix} 0.066 & 0.030 & 0.021 \\ 0.030 & 0.066 & 0.030 \\ 0.021 & 0.030 & 0.066 \end{matrix} \right)$ |
| **Unstructured** | - | - | $\left( \begin{matrix} 0.092 & 0.050 & 0.047 \\ 0.050 & 0.086 & 0.072 \\ 0.047 & 0.072 & 0.093 \end{matrix} \right)$ | - | - | $\left( \begin{matrix} 0.049 & 0.012 & 0.026 \\ 0.012 & 0.054 & 0.044 \\ 0.026 & 0.044 & 0.095 \end{matrix} \right)$ |

WPICC: within-period ICC, CAC: Cluster autocorrelation coefficient

^a^ Each cell [i, j] represent correlation between two within-cluster individuals collected in different i and j periods.

**Table S2 Comparisons of intervention effect estimates using mixed-effects logistic regression models assuming five different correlation structures as well as assuming robust variance estimators with exchangeable correlation - adjusted for prespecified covariates**

| **Intervention** | **Model** | **12-MONTH** | | | | **18-MONTH** | | | | **24-MONTH** | | | |
| --- | --- | --- | --- | --- | --- | --- | --- | --- | --- | --- | --- | --- | --- |
|  |  | **OR** | **95%CI** | **SE** | **P-value** | **OR** | **95%CI** | **SE** | **P-value** | **OR** | **95%CI** | **SE** | **P-value** |
| **Intervention 2** | *UN* | 0.648 | [0.424, 0.991] | 0.2165 | 0.0453 | 0.931 | [0.676, 1.281] | 0.1630 | 0.6610 | 0.754 | [0.481, 1.18] | 0.2287 | 0.2162 |
|  | *TOEP* | 0.689 | [0.457, 1.038] | 0.2091 | 0.0750 | 0.990 | [0.674, 1.455] | 0.1963 | 0.9612 | 0.792 | [0.538, 1.167] | 0.1977 | 0.2383 |
|  | *EXP* | 0.695 | [0.461, 1.046] | 0.2090 | 0.0813 | 0.991 | [0.675, 1.456] | 0.1961 | 0.9646 | 0.781 | [0.531, 1.151] | 0.1975 | 0.2115 |
|  | *NE* | 0.648 | [0.424, 0.991] | 0.2165 | 0.0453 | 0.931 | [0.676, 1.281] | 0.1630 | 0.6610 | 0.754 | [0.481, 1.18] | 0.2287 | 0.2162 |
|  | *EXCH* | 0.695 | [0.493, 0.978] | 0.1746 | 0.0369 | 1.000 | [0.724, 1.382] | 0.1649 | 0.9989 | 0.794 | [0.574, 1.098] | 0.1652 | 0.1626 |
|  | *RVE* | 0.697 | [0.413, 1.175] | 0.2667 | 0.1754 | 1.000 | [0.692, 1.445] | 0.1876 | 0.9990 | 0.796 | [0.469, 1.352] | 0.2702 | 0.3984 |
| **Intervention 3** | *UN* | 0.650 | [0.427, 0.991] | 0.2148 | 0.0453 | 0.768 | [0.56, 1.053] | 0.1608 | 0.1008 | 0.986 | [0.631, 1.54] | 0.2275 | 0.9509 |
|  | *TOEP* | 0.655 | [0.436, 0.986] | 0.2084 | 0.0427 | 0.771 | [0.526, 1.131] | 0.1953 | 0.1831 | 0.990 | [0.674, 1.454] | 0.1961 | 0.9586 |
|  | *EXP* | 0.660 | [0.439, 0.993] | 0.2082 | 0.0462 | 0.771 | [0.526, 1.13] | 0.1952 | 0.1818 | 0.977 | [0.665, 1.434] | 0.1959 | 0.9034 |
|  | *NE* | 0.650 | [0.427, 0.991] | 0.2148 | 0.0453 | 0.768 | [0.56, 1.053] | 0.1608 | 0.1008 | 0.986 | [0.631, 1.54] | 0.2275 | 0.9509 |
|  | *EXCH* | 0.631 | [0.448, 0.889] | 0.1745 | 0.0084 | 0.775 | [0.562, 1.067] | 0.1633 | 0.1180 | 0.964 | [0.7, 1.327] | 0.1631 | 0.8217 |
|  | *RVE* | 0.633 | [0.389, 1.028] | 0.2477 | 0.0648 | 0.775 | [0.531, 1.132] | 0.1932 | 0.1871 | 0.965 | [0.613, 1.519] | 0.2315 | 0.8768 |
| **Intervention 4** | *UN* | 0.448 | [0.292, 0.687] | 0.2184 | 0.0002 | 0.642 | [0.466, 0.883] | 0.1627 | 0.0064 | 0.435 | [0.276, 0.684] | 0.2314 | 0.0003 |
|  | *TOEP* | 0.468 | [0.309, 0.71] | 0.2119 | 0.0003 | 0.671 | [0.456, 0.986] | 0.1965 | 0.0421 | 0.449 | [0.303, 0.666] | 0.2008 | <.0001 |
|  | *EXP* | 0.474 | [0.313, 0.717] | 0.2117 | 0.0004 | 0.673 | [0.458, 0.988] | 0.1962 | 0.0432 | 0.444 | [0.3, 0.658] | 0.2006 | <.0001 |
|  | *NE* | 0.448 | [0.292, 0.687] | 0.2184 | 0.0002 | 0.664 | [0.453, 0.975] | 0.1957 | 0.0367 | 0.435 | [0.276, 0.684] | 0.2314 | 0.0003 |
|  | *EXCH* | 0.457 | [0.322, 0.65] | 0.1789 | <.0001 | 0.672 | [0.486, 0.929] | 0.1653 | 0.0161 | 0.449 | [0.323, 0.624] | 0.1683 | <.0001 |
|  | *RVE* | 0.460 | [0.288, 0.735] | 0.2392 | 0.0012 | 0.673 | [0.480, 0.945] | 0.1729 | 0.0220 | 0.451 | [0.291, 0.700] | 0.2242 | 0.0004 |

OR: Odds ratio, CI: Confidence interval, SE: Standard error, UN: Unstructured, TOEP: Toeplitz, EXP: Exponential decay, NE: Nested exchangeable, EXCH: Exchangeable, RVE: Robust variance estimation

**Table S3 Comparisons of intervention effect estimates using mixed-effects logistic regression models assuming five different correlation structures as well as assuming robust variance estimators with exchangeable correlation - unadjusted for prespecified covariates**

| **Intervention** | **Model** | **12-MONTH** | | | | **18-MONTH** | | | | **24-MONTH** | | | |
| --- | --- | --- | --- | --- | --- | --- | --- | --- | --- | --- | --- | --- | --- |
|  |  | **OR** | **95%CI** | **SE** | **P-value** | **OR** | **95%CI** | **SE** | **P-value** | **OR** | **95%CI** | **SE** | **P-value** |
| **Intervention 2** | *UN* | 0.674 | [0.362, 1.255] | 0.3174 | 0.2135 | 0.952 | [0.575, 1.576] | 0.2570 | 0.8494 | 0.755 | [0.423, 1.346] | 0.2951 | 0.3405 |
|  | *TOEP* | 0.666 | [0.374, 1.187] | 0.2946 | 0.1682 | 0.948 | [0.542, 1.659] | 0.2854 | 0.8521 | 0.747 | [0.426, 1.309] | 0.2864 | 0.3074 |
|  | *EXP* | 0.661 | [0.371, 1.178] | 0.2948 | 0.1604 | 0.949 | [0.542, 1.662] | 0.2856 | 0.8556 | 0.751 | [0.428, 1.317] | 0.2866 | 0.3171 |
|  | *NE* | 0.669 | [0.377, 1.188] | 0.2929 | 0.1702 | 0.944 | [0.541, 1.646] | 0.2837 | 0.8385 | 0.757 | [0.433, 1.322] | 0.2848 | 0.3276 |
|  | *EXCH* | 0.668 | [0.396, 1.127] | 0.2669 | 0.1302 | 0.958 | [0.575, 1.597] | 0.2606 | 0.8701 | 0.760 | [0.456, 1.267] | 0.2607 | 0.2931 |
|  | *RVE* | 0.669 | [0.337, 1.325] | 0.3489 | 0.2490 | 0.958 | [0.552, 1.661] | 0.2811 | 0.8774 | 0.761 | [0.406, 1.428] | 0.3208 | 0.3954 |
| **Intervention 3** | *UN* | 0.573 | [0.307, 1.069] | 0.3183 | 0.0801 | 0.668 | [0.403, 1.107] | 0.2578 | 0.1171 | 0.847 | [0.475, 1.512] | 0.2954 | 0.5745 |
|  | *TOEP* | 0.566 | [0.317, 1.011] | 0.2956 | 0.0545 | 0.658 | [0.376, 1.153] | 0.2861 | 0.1439 | 0.840 | [0.479, 1.474] | 0.2866 | 0.5439 |
|  | *EXP* | 0.564 | [0.316, 1.006] | 0.2958 | 0.0526 | 0.659 | [0.376, 1.155] | 0.2864 | 0.1454 | 0.845 | [0.482, 1.483] | 0.2869 | 0.5575 |
|  | *NE* | 0.567 | [0.319, 1.009] | 0.2940 | 0.0538 | 0.658 | [0.376, 1.149] | 0.2846 | 0.1407 | 0.852 | [0.487, 1.49] | 0.2851 | 0.5753 |
|  | *EXCH* | 0.548 | [0.324, 0.927] | 0.2683 | 0.0250 | 0.670 | [0.401, 1.117] | 0.2611 | 0.1246 | 0.836 | [0.501, 1.394] | 0.2609 | 0.4920 |
|  | *RVE* | 0.550 | [0.275, 1.1] | 0.3539 | 0.0908 | 0.671 | [0.367, 1.226] | 0.3075 | 0.1940 | 0.837 | [0.432, 1.623] | 0.3379 | 0.5981 |
| **Intervention 4** | *UN* | 0.461 | [0.247, 0.861] | 0.3190 | 0.0152 | 0.652 | [0.394, 1.078] | 0.2566 | 0.0956 | 0.437 | [0.244, 0.781] | 0.2967 | 0.0052 |
|  | *TOEP* | 0.455 | [0.255, 0.813] | 0.2963 | 0.0079 | 0.649 | [0.371, 1.134] | 0.2849 | 0.1291 | 0.432 | [0.246, 0.76] | 0.2881 | 0.0036 |
|  | *EXP* | 0.453 | [0.253, 0.809] | 0.2965 | 0.0075 | 0.650 | [0.372, 1.137] | 0.2852 | 0.1311 | 0.434 | [0.247, 0.764] | 0.2883 | 0.0038 |
|  | *NE* | 0.456 | [0.256, 0.813] | 0.2947 | 0.0078 | 0.647 | [0.371, 1.127] | 0.2833 | 0.1237 | 0.439 | [0.25, 0.769] | 0.2865 | 0.0040 |
|  | *EXCH* | 0.449 | [0.265, 0.761] | 0.2692 | 0.0030 | 0.659 | [0.396, 1.097] | 0.2603 | 0.1088 | 0.441 | [0.264, 0.737] | 0.2622 | 0.0018 |
|  | *RVE* | 0.451 | [0.237, 0.859] | 0.3286 | 0.0154 | 0.660 | [0.395, 1.104] | 0.2624 | 0.1135 | 0.443 | [0.238, 0.827] | 0.3180 | 0.0105 |

OR: Odds ratio, CI: Confidence interval, SE: Standard error, UN: Unstructured, TOEP: Toeplitz, EXP: Exponential decay, NE: Nested exchangeable, EXCH: Exchangeable, RVE: Robust variance estimation

**Figure S1 Comparison of intervention effect estimates and 95% Confidence Intervals using unadjusted mixed-effects logistic regression models assuming five different correlation structures as well as assuming robust variance estimators with exchangeable correlation**

**
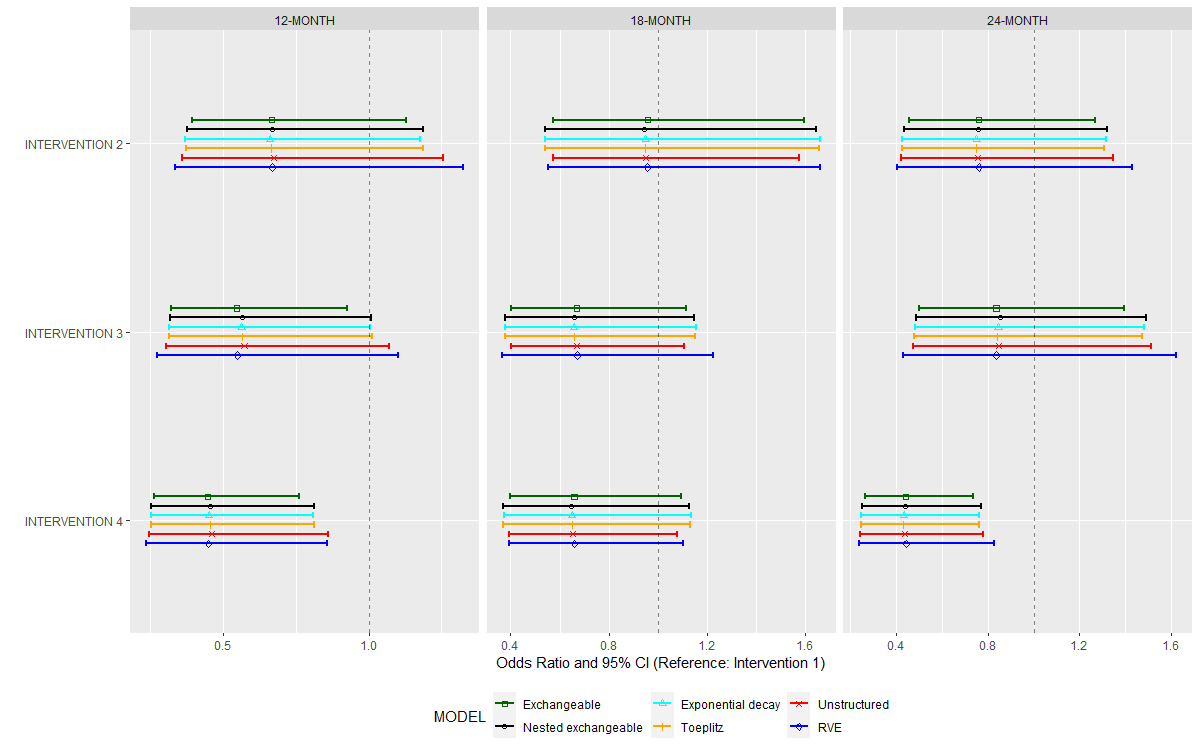
**
