# Supplementary material for: Accounting for complex intracluster correlations in longitudinal cluster randomized trials: a case study in malaria vector control
Source: BMC Med Res Methodol. 2023 Mar 17;23:64. doi: 10.1186/s12874-023-01871-2 (PMC10021932; doi:10.1186/s12874-023-01871-2)
Supplement: Supplementary file 3 — Additional file 3. [file 12874_2023_1871_MOESM3_ESM.docx]

**Additional file 3: Proof-of-concept simulation**

We conducted a proof-of-concept simulation study to demonstrate the impact of over- or under-specifying the correlation structure within a linear mixed-effects regression analysis for a parallel arm cluster randomized trial. For reference, the correctly specified correlation structure was also fitted. For simplicity, we assumed a continuous outcome with the parameter of interest the average treatment effect over time. For each trial configuration, we simulated 2,000 datasets, leading to a Monte Carlo Standard Error (MCSE) $\pm0.5\%$ for the coverage probability. The data generation and analyses were conducted on the Compute Canada High-Performance Computing Cluster with R version 4.0.2.

1. **Data generation and analysis: Model under-specification**

First, we generated data under a complex correlation structure (unstructured) and investigated the impact of fitting a simpler correlation structure to the data. The simulation parameters were obtained from the example trial and are summarized in Table S1 below. We generated data from the following model:

$$y_{ijk}= \beta_{j}+ \delta X_{ij}+\gamma_{ij}+ \varepsilon_{ijk}$$

where $y_{ijk}$ is the observation from the $k$-th individual in the $i$-th cluster and $j$-th period; $\beta_{j}$ is the categorical time effect in the $j$-th period (assuming $\beta_{j}=j$); $X_{ij}$ is the binary treatment indicator in the $i$-th cluster and $j$-th period. The vector of random cluster-by-period effects in each cluster is assumed to follow $\boldsymbol{\gamma}_{\boldsymbol{i}}=\left( \gamma_{i1},\ldots,\gamma_{iJ} \right)^{'}\sim N\left( 0,\sigma_{\gamma}^{2}\tilde{Z} \right)$. We considered three possible variance-covariance matrices for the random effects (see Table S1). The first matrix corresponds to a scenario where the within-period ICC increases linearly over successive periods, the second matrix corresponds to a scenario where the within-period ICCs changes in any direction, and the third matrix corresponds to a scenario where the within-period ICC decreases linearly over successive periods.

The simulated data were then analyzed using linear mixed-effects regression with the following correlation structures:

1. Exchangeable
2. Exchangeable with robust variance estimator (RVE)
3. Nested exchangeable
4. Exponential decay
5. Toeplitz
6. Unstructured (true model)

For the RVE, we used the estimator that approximates the leave-one-cluster-out jackknife variance estimator, which has been recommended by Bell and McCaffrey.^1^

**Table S1: Simulation parameters for investigating the implications of model under-specification**

| **Parameter** | **Value** |
| --- | --- |
| Number of clusters | 84 |
| Number of periods | 4 (one baseline + three follow-ups) |
| Cluster period sizes | 45 |
| Treatment effect size | 0 |
| Variance of individual error term | 0.2 |
| Variance-covariance matrix for $\boldsymbol{\gamma}_{\boldsymbol{i}}$, i.e., $\sigma_{\gamma}^{2}\tilde{Z}$ | $\left( 1 \right)\left( \begin{matrix} 0.100 & 0.090 & 0.080 & 0.050 \\ 0.090 & 0.150 & 0.100 & 0.125 \\ 0.080 & 0.100 & 0.200 & 0.090 \\ 0.050 & 0.125 & 0.090 & 0.250 \end{matrix} \right)$  $\left( 2 \right)\left( \begin{matrix} 0.100 & 0.090 & 0.080 & 0.050 \\ 0.090 & 0.200 & 0.100 & 0.125 \\ 0.080 & 0.100 & 0.150 & 0.090 \\ 0.050 & 0.125 & 0.090 & 0.250 \end{matrix} \right)$  $(3)\left( \begin{matrix} 0.250 & 0.150 & 0.120 & 0.110 \\ 0.150 & 0.200 & 0.125 & 0.100 \\ 0.120 & 0.125 & 0.150 & 0.090 \\ 0.110 & 0.100 & 0.090 & 0.100 \end{matrix} \right)$ |

1. **Data generation and analysis: Model over-specification**

Next, we generated data under a simpler correlation structure and investigated the impact of fitting a more complex correlation. The simulation parameters are summarized in Table S2 below. We generated data from the following model:

$$y_{ijk}=\beta_{j}+ \delta X_{ij}+\alpha_{i}+ \varepsilon_{ijk}$$

where $y_{ijk}$ is the observation from the $k$-th individual in the $i$-th cluster and $j$-th period; $\beta_{j}$ is the categorical time effect in the $j$-th period (assuming $\beta_{j}=j$); $X_{ij}$ is the binary treatment indicator in the $i$-th cluster and $j$-th period. The term $\alpha_{i}\sim N\left( 0,\sigma_{\alpha}^{2} \right)$ represents the random intercept for clusters. We considered two possible variances for the random intercept (see Table S2), which yield an Intracluster Correlation Coefficient (ICC) of 0.05 and 0.1.

The simulated data then were then analyzed using linear mixed-effects regression with the following correlation structures:

1. Exchangeable (true model)
2. Exchangeable with robust variance estimator (RVE)
3. Exponential decay
4. Toeplitz
5. Unstructured

**Table S2: Simulation parameters for investigating the implications of model over-specification**

| **Parameter** | **Value** |
| --- | --- |
| Number of clusters | 84 |
| Number of periods | 4 (one baseline + three follow-ups) |
| Number of cluster period sizes | 45 |
| Treatment effect size | 0 |
| Variance of individual error term | 0.20 |
| Variance of random intercept for clusters | (1) 0.011 (corresponding to an ICC of 0.05)  (2) 0.022 (corresponding to an ICC of 0.10) |

1. **Performance measures**

For each fitted model, we measured the following quantities to assess the performance of the model.

**Table S3: Performance measures assessed in the simulation study**

| **Performance measures** | **Definition** | **Estimates** |
| --- | --- | --- |
| **Bias** | $E\left[ \hat{\theta} \right]-\theta$ | $\frac{1}{n_{sim}}\sum_{i=1}^{n_{sim}} \hat{\theta_{i}}-\theta$ |
| **Coverage** | $P(\hat{\theta_{low}}<\theta<\hat{\theta_{upp}})$ | $\frac{1}{n_{sim}}\sum_{i=1}^{n_{sim}} I(\hat{\theta_{low}}<\theta<\hat{\theta_{upp}})$ |
| **Average Model Standard Error (SE)** | $\sqrt{E[\hat{Var}(\hat{\theta})]}$ | $\sqrt{\frac{1}{n_{sim}}\sum_{i=1}^{n_{sim}} \hat{Var}(\hat{\theta_{i}})}$ |
| **Empirical SE** | $\sqrt{Var(\hat{\theta})}$ | $\sqrt{\frac{1}{n_{sim}}\sum_{i=1}^{n_{sim}} {(\hat{\theta_{i}}-\bar{\theta})}^{2}}$ |
| **Relative error of model-based SE** | *100(* $\frac{Model SE}{Empirical SE}-1$*)* | *100*$(\frac{\hat{Model SE}}{\hat{Empirical SE}}-1)$ |

*Source: Morris et al^2^

$\theta$ is the parameter of interest; $\hat{\theta}$ is the estimate; $\bar{\theta}$ is the average estimate over all simulation runs

1. **Results: Model under-specification**

In this section we report the results from the simulation scenarios where the data were generated from an unstructured model and analyzed using a simpler model. Simulation results are presented in Table S4, Table S5, Table S6 and Table S7.

Although random effects were mis-specified, the estimated treatment effects were unbiased in all scenarios examined (Table S4). When the model was under-specified, we found that the bias of the model-based standard error was as large as 75% under the exchangeable model, leading to a 95% coverage probability around 40%; when we allowed for a decay, the coverage probabilities were closer to, but still below, the nominal level (Tables S5 and S7). Depending on how the within-period and between-period ICCs changed over time, the exponential decay and Toeplitz models returned coverage probabilities either below or above 95%, and the estimated model-based SE were either smaller or larger than the model-based SE from the unstructured model (Table S6). Adding robust variance estimators (RVEs) to the exchangeable model maintained the validity of statistical inferences while a loss in efficiency was noted (the SE was inflated by 7% to 49%).

**Table S4: Bias of the point estimate for the treatment effect**

|  | **(1) Within-period ICC increases linearly** | **(2) Within-period ICC changes in any direction** | **(3) Within-period ICC decreases linearly** |
| --- | --- | --- | --- |
| **Specified analysis model** |  |  |  |
| **Exchangeable** | 0.001 | -0.001 | -0.003 |
| **Nested exchangeable** | 0.001 | -0.001 | -0.001 |
| **Exponential decay** | 0.001 | -0.001 | -0.001 |
| **Toeplitz** | 0.001 | -0.001 | 0.000 |
| **Unstructured** | 0.001 | -0.001 | 0.000 |

**Table S5: Coverage of the 95% confidence interval for the treatment effect**

|  | **(1) Within-period ICC increases linearly** | **(2) Within-period ICC changes in any direction** | **(3) Within-period ICC decreases linearly** |
| --- | --- | --- | --- |
| **Specified analysis model** |  |  |  |
| **Exchangeable** | 0.682 | 0.701 | 0.400 |
| **Exchangeable with RVE** | 0.947 | 0.951 | 0.950 |
| **Nested exchangeable** | 0.936 | 0.929 | 0.947 |
| **Exponential decay** | 0.950 | 0.932 | 0.961 |
| **Toeplitz** | 0.961 | 0.961 | 0.937 |
| **Unstructured** | 0.950 | 0.948 | 0.950 |

**Table S6: Ratio of standard errors (relative to the true model)**

|  | **(1) Within-period ICC increases linearly** | **(2) Within-period ICC changes in any direction** | **(3) Within-period ICC decreases linearly** |
| --- | --- | --- | --- |
| **Specified analysis model** |  |  |  |
| **Exchangeable** | 0.549 | 0.542 | 0.366 |
| **Exchangeable with RVE** | 1.082 | 1.069 | 1.487 |
| **Nested exchangeable** | 0.969 | 0.956 | 1.091 |
| **Exponential decay** | 1.041 | 1.027 | 1.236 |
| **Toeplitz** | 1.060 | 1.057 | 0.937 |
| **Unstructured** | 1.000 | 1.000 | 1.000 |

**Table S7: Relative error of model-based standard errors (%)**

|  | **(1) Within-period ICC increases linearly** | **(2) Within-period ICC changes in any direction** | **(3) Within-period ICC decreases linearly** |
| --- | --- | --- | --- |
| **Specified analysis model** |  |  |  |
| **Exchangeable** | -49.0 | -48.1 | -74.6 |
| **Exchangeable with RVE** | -0.5 | 2.5 | 3.3 |
| **Nested exchangeable** | -8.0 | -7.6 | -2.4 |
| **Exponential decay** | -2.4 | -6.2 | 5.4 |
| **Toeplitz** | 3.1 | 3.1 | 5.7 |
| **Unstructured** | -2.0 | -0.7 | 0.4 |

1. **Results: Model over-specification**

In this section we report the results from the simulation scenarios where data were generated under a simpler correlation structure and analyzed using a more complex correlation. where the data were generated from exchangeable models and analyzed using complex models. Simulation results are presented in Table S8, Table S9, Table S10 and Table S11. We also present the model non-convergence rates in Table S12.

Although random effects were mis-specified, the estimated treatment effects were unbiased in all scenarios examined (Table S8). When the model was over-specified, we found that the nominal level of coverage was maintained (Table S9). The average model-based standard error was slightly inflated (by no more than 7%), indicating a potential loss of efficiency (Table S10). It is worthwhile noting that over-specification led to substantial non-convergence: among all simulation runs, more than 86% of Toeplitz and unstructured models failed to converge (Table S12). In this case, the exchangeable model with RVE was a good solution with minimal loss of efficiency from the use of RVE.

**Table S8: Bias of the point estimate for the treatment effect**

|  | **(1)** **ICC = 0.05** | **(2) ICC=0.10** |
| --- | --- | --- |
| **Specified analysis model** |  |  |
| **Exchangeable** | 0.000 | 0.000 |
| **Exponential decay** | 0.000 | 0.000 |
| **Toeplitz** | 0.000 | -0.001 |
| **Unstructured** | 0.000 | 0.000 |

**Table S9: Coverage of the 95% confidence interval for the treatment effect**

|  | **(1)** **ICC = 0.05** | **(2) ICC=0.10** |
| --- | --- | --- |
| **Specified analysis model** |  |  |
| **Exchangeable** | 0.948 | 0.949 |
| **Exchangeable with RVE** | 0.945 | 0.946 |
| **Exponential decay** | 0.955 | 0.952 |
| **Toeplitz** | 0.957 | 0.950 |
| **Unstructured** | 0.945 | 0.950 |

**Table S10: Ratio of standard errors (relative to the true model)**

|  | **(1)** **ICC = 0.05** | **(2) ICC=0.10** |
| --- | --- | --- |
| **Specified analysis model** |  |  |
| **Exchangeable** | 1.000 | 1.000 |
| **Exchangeable with RVE** | 1.009 | 1.009 |
| **Exponential decay** | 1.020 | 1.020 |
| **Toeplitz** | 1.055 | 1.057 |
| **Unstructured** | 1.053 | 1.062 |

**Table S11: Relative error of model-based standard error (%)**

|  | **(1)** **ICC = 0.05** | **(2) ICC=0.10** |
| --- | --- | --- |
| **Specified analysis model** |  |  |
| **Exchangeable** | -2.7 | -2.5 |
| **Exchangeable with RVE** | -1.8 | -1.6 |
| **Exponential decay** | -0.4 | -1.0 |
| **Toeplitz** | 4.4 | 4.9 |
| **Unstructured** | -3.4 | -3.6 |

**Table S12: Percentage of models failing to converge (%)**

|  | **(1)** **ICC = 0.05** | **(2) ICC=0.10** |
| --- | --- | --- |
| **Specified analysis model** |  |  |
| **Exchangeable** | 0.0 | 0.0 |
| **Exponential decay** | 8.2 | 7.51 |
| **Toeplitz** | 87.0 | 88.1 |
| **Unstructured** | 89.1 | 88.0 |

**6. Summary**

To summarize, our limited simulation study suggests that fitting more flexible correlation structures may be required to maintain statistical validity; in cases of non-convergence, the exchangeable model with RVEs is a reasonable solution, although the loss of efficiency can be large in some cases.

**References**

1. Bell RM, McCaffrey DF. Bias reduction in standard errors for linear regression with multi-stage samples. *Survey Methodology*.

2. Morris TP, White IR, Crowther MJ. Using simulation studies to evaluate statistical methods. *Statistics in Medicine* 2019; 38: 2074–2102.
